# Evaluation of Surrogate Endpoints Using Information-Theoretic Measure of Association Based on Havrda and Charvat Entropy

**DOI:** 10.3390/math10030465

**Published:** 2022-01-31

**Authors:** María del Carmen Pardo, Qian Zhao, Hua Jin, Ying Lu

**Affiliations:** 1 Department of Statistics and O.R., Complutense University of Madrid, 28040 Madrid, Spain;; 2 Department of Epidemiology and Health Statistics, School of Public Health, Guangzhou Medical University, Guangzhou 510260, China;; 3 Department of Probability and Statistics, School of Mathematics, South China Normal University, Guangzhou 510631, China;; 4 Department of Biomedical Data Science, Stanford University, San Francisco, CA 94305-5464, USA

**Keywords:** surrogate endpoint, information theory, Havrda and Charvat entropy, mutual information, clinical trial design

## Abstract

Surrogate endpoints have been used to assess the efficacy of a treatment and can potentially reduce the duration and/or number of required patients for clinical trials. Using information theory, Alonso et al. (2007) proposed a unified framework based on Shannon entropy, a new definition of surrogacy that departed from the hypothesis testing framework. In this paper, a new family of surrogacy measures under Havrda and Charvat (H-C) entropy is derived which contains Alonso’s definition as a particular case. Furthermore, we extend our approach to a new model based on the information-theoretic measure of association for a longitudinally collected continuous surrogate endpoint for a binary clinical endpoint of a clinical trial using H-C entropy. The new model is illustrated through the analysis of data from a completed clinical trial. It demonstrates advantages of H-C entropy-based surrogacy measures in the evaluation of scheduling longitudinal biomarker visits for a phase 2 randomized controlled clinical trial for treatment of multiple sclerosis.

## 1. Introduction

Surrogate endpoints which can be observed earlier, easier, possibly repeated, or are cost-saving, have been used to replace clinical endpoints in clinical trials. For example, total tumor response rate and progression–free survival have been used in phase II and phase III cancer clinical trials as surrogate endpoints for overall survival, which often requires a longer trial duration to achieve adequate statistical power. The United States Food and Drug Administration (USFDA) has accepted the use of surrogate endpoints in regulatory reviews of new drug applications [1]. Most cancer drug approvals (55 of 83 (66%)) between 2009 and 2014 by the USFDA have used at least one surrogate endpoint [2].

A motivative example is to use biomarkers in phase II cancer trials. Non-randomized single arm or randomized parallel clinical trials are used to evaluate signal of efficacy for a new drug. A binary response status, such as the total response based on the RECIST criterion [3], or a continuous response in change of tumor sizes [4] are common primary endpoints. For molecular-targeted drugs or immune oncology therapies, various serum, tissue, or imaging biomarkers are being developed to assess if the targeted pathways have been activated. These biomarkers are usually continuous, can be measured repeatedly, and their changes should proceed to a clinical response. However, the activation of a targeted pathway doesn’t necessarily imply the response to the treatment. Various questions have been raised about the utility of such biomarkers in phase II trials [5,6].

Surrogate endpoints provide the convenience to speed up the clinical trial [7], but may not represent the actual outcomes well regarding the benefit of therapy [8]. For instance, bevacizumab was approved in metastatic breast cancer based on the surrogate outcome and was later withdrawn for failing to confirm a survival benefit [5]. How to evaluate the therapy benefit between surrogates, denoted as *S* and the true clinical endpoint outcome, denoted as *T*, remains a scientific challenge.

There are a lot of successful statistical methods and measures to assess surrogate endpoints. One method to validate surrogate endpoints is to evaluate their correlation with clinically meaningful endpoints through meta-analyses [9]. Only 11 of 89 (12%) studies had found high correlation (r ≥ 0.85), and nine (10%) showed a moderate-only correlation (r > 0.7 to r < 0.85) between surrogates and endpoints [7], suggesting that the strength of surrogates in clinical practice is often unknown or weak.

The landmark paper by Prentice [8] proposed operational criteria for the identification of valid surrogate endpoints. A sufficient condition for an endpoint *S*, as a valid surrogate of a primary clinical endpoint *T*, in the evaluation of a treatment, denoted as *Z*, is that the random vector (*Z*, *S*, *T*) forms a Markov chain *Z* → *S* → *T*, i.e., conditioning on *S*, *Z* and *T* are independent from each other. This condition led to parametric and non-parametric approaches to quantify the proportion of the treatment effect on *T* that is explained by the treatment effect on the *S* [9–13]. Other proposed quantities to assess the utility of a surrogate endpoint include dissociative effects, associative effects, average causal necessity, average causal sufficiency, causal effect predictiveness surface, and principle surrogate, etc. [9,14–22].

Buyse and Molenberghs [23] suggested two quantities to validate a surrogate endpoint: the relative effect which related the treatment effect on the primary outcome to that on the surrogate at the population level, and the adjusted association, which quantified the association between the primary outcome and surrogate marker after adjusting for the treatment at the individual level. These methods assumed that information regarding the surrogate and true endpoints was available from a single-trial surrogate evaluation method. They focused only on the validity, whereas the general association of the last one was related to the efficiency of a surrogate marker. Using an information-theoretic approach, Alonso and Molenberghs [24] and Pryseley et al. [25] redefined surrogacy in terms of the information content that *S* provides with respect to *T*. Using notations from Pryseley et al [25], let *S* be a continuous surrogate random variable and *T* be the continuous targeted clinical endpoint of interest. We use *f*(·) to denote the density function. The Shannon entropy functions for *T* and the conditional variable *T*|*S* are denoted as *h*(*T*) and *h*(*T*|*S*), respectively, where *h*(*T*) = *E*[−*logf*(*T*)] and *h*(*T*|*S*) = *E*[−*logf*(*T*|*S*)]. The corresponding entropy power functions are $EP_{h}(T)=e^{\frac{2}{n}h(T)}/(2\pi e)$ and $EP_{h}(T|S)=e^{\frac{2}{n}h(T|S)}/(2\pi e)$. An information theoretic measure of association (ITMA) is defined by Alonso and colleagues as the proportion of uncertainty reduction measured by the entropy power function for *T*|*S* in reference to *T*:

(1) $$R_{h}^{2}=\frac{EP_{h}(T)-EP_{h}(T|S)}{EP_{h}(T)}=1-e^{-2I(T,S)}$$

Here *I*(*T*, *S*) = *E*[−*logf*(*T*)] – *E*[−*logf*(*T*|*S*)] is the mutual information. If *S* is a good surrogate for *T*, uncertainty about the effect on *T* is reduced by knowing the knowledge of the effect on *S*. There are some useful properties as described by Alonso and Molenberghs [24]:

1. $0\leq R_{h}^{2}\leq 1$
2. $R_{h}^{2}=0$, if and only if (*T*, *S*) are independent
3. $R_{h}^{2}$ is symmetric in (*T*, *S*)
4. $R_{h}^{2}$ is invariant under bijective transformations of *T* and *S*
5. When $R_{h}^{2}\to 1$ for continuous models, there is usually a deterministic relationship in the distribution of (*T*, *S*), that is, often *T* = φ(*S*).
6. When T is a discrete random variable, $R_{h}^{2}\leq 1-e^{-2h(T)}$. So they propose to use $R_{h,max}^{2}=R_{h}^{2}/\left(1-e^{-2h(T)}\right)$ as a modified ITMA.

A good surrogacy should have a high $R_{h}^{2}$. The beauty of the informatic-theoretical framework is that it moves away from hypothesis testing and provides a quantitative measure of surrogacy. Thus, if there are two surrogate endpoints, *S*_1_ and *S*_2_, we can compare their utility as surrogacy endpoints based on the value of $R_{h}^{2\prime }\text{s}$.

Multiple authors have provided examples of this approach and demonstrated applications for situations when *S* and *T* are both binary, continuous, longitudinal, and time-to-event random variables as well as ordinal outcomes [24–28].

In this paper, we present two new results on the topic of surrogate endpoints based on information-theoretic measure of association (ITMA). First, we extend the ITMA construction based on Shannon entropy to a construction based on Havrda and Charvat (H-C) entropy [29]. The extension is motivated by the general existence of the H-C entropy. Explicit expressions as well as the properties of ITMA in different situations based on H-C entropy are presented. Second, we extend the H-C ITMA model to a longitudinally collected continuous surrogate endpoint for a binary clinical endpoint of a clinical trial. Then, the benefit of *S* is evaluated with the ITMA [24,25]. The current work focuses on a single trial surrogacy and its extension to a meta-analytic framework will need further development.

The paper is organized in the following structure. In Section 2, we give an example of when the Shannon entropy cannot be defined, thus the surrogacy by Alonso and Molenberghs under the information theoretical framework will not work [24]. We then prove the existence of H-C entropy under general conditions. Therefore, a family of surrogacy measures based on ITMA of H-C entropy is defined. An explicit formula is obtained for the following situations: binary-binary, continuous–continuous and binary-continuous. In Section 3, we extend a longitudinal linear random effects model for the longitudinally collected surrogate marker and a probit regression model for a binary primary endpoint in clinical trials. An application using H-C entropy in selecting times to collect surrogate measures is presented using data from a completed clinical trial. Finally, Section 4 presents discussions and a conclusion. R-Programs that generated re-sults for tables in Section 3 are presented in the Appendix A.

## 2. Extension of ITMA Surrogacy from Shannon Entropy to Havrda-Charvat Entropy

Why should we consider the extension? While Shannon’s entropy is adequate in most applications, there are cases when a Shannon’s entropy function doesn’t exist, and thus the ITMA cannot be properly calculated. We give an example in the following.

### Example 1.

*Let X be the random variable with heavy tails*. *Its density function is*

(2) $$f(x)=\{\begin{array}{ll} \frac{1}{\sqrt{2\pi }}e^{-\frac{x^{2}}{2}}, & |x|\leq c_{1}\\ \frac{c_{2}}{|x|{\left(\text{log}|x|\right)}^{2}}, & |x|>c_{1} \end{array}$$

where *c*_1_ ≈ 1.44 and *c*_2_ ≈ 0.027 are chosen so that it is a continuous function with a heavy tail. The Shannon entropy *h*(*X*) for *X* is infinity.

One way to make ITMA work is to use the Havrda-Charvat entropy [29], a generalization of entropy function that contains the Shannon entropy as its special case. Mathematically, Havrda-Charvat entropy is defined as follows:

(3) $$HC_{\alpha }(X)=\int \varphi_{\alpha }(f(x))f(x)dx.$$

where

(4) $$\varphi_{\alpha }(x)=\{\begin{array}{cc} \left(x^{\alpha -1}-1\right)/(1-\alpha ) & \alpha \neq 1\\ -log(x) & \alpha =1 \end{array}$$

It is easy to see that *HC*_1_(*X*) = *h*(*X*).

### Proposition 1.

*Under a mild regular condition that the density function for X is bounded*, *HC*_*α*_(*X*) *can always exist for an α* > 1.

### Proof.

We only need to prove that for a bounded density function *f*(*x*) by a constant *K*>0, $\int {\left(f(x)\right)}^{a}dx<+\infty$.

Let *W* = {*x*: *f*(*x*) > 1}, then it follows from the fact that

$$1=\int f(x)dx=\int_{W}f(x)dx+\int_{W^{C}}f(x)dx\geq m(W)+\int_{W^{C}}f(x)dx$$

that *m*(*W*) ≤ 1 and $\int_{W^{C}}f(x)dx\leq 1$, where *m*(*W*) is the probability of *W*. Therefore,

$$\int {\left(f(x)\right)}^{\alpha }dx=\int_{W}{\left(f(x)\right)}^{\alpha }dx+\int_{W^{C}}{\left(f(x)\right)}^{\alpha }dx\leq K^{\alpha -1}m(W)\int_{W^{C}}f(x)dx<+\infty$$

Because Shannon’s entropy is a special case of H-C entropy and H-C entropy always exists with proper choice of *α*, ITMA of H-C entropy should be a more flexible way as a surrogacy measure for more distribution families. Different from Shannon’s entropy, H-C entropy satisfies a non-additive property such that *HC*_*α*_(*T*, *S*) = *HC*_*α*_(*T*) + *HC*_*α*_(*S*) + (1 − *α*)*HC*_*α*_(*T*)*HC*_*α*_(*S*), when *T* and *S* are independent. In general, the non-additive measures of entropy find justifications in many biological and chemical phenomena [30]. While H-C entropy has been used in quantum physics [31] and medical imaging research [32], it has not yet been used to describe the endpoint surrogacy for clinical trials.

To extend H-C entropy to measure the endpoint surrogacy for trials, we define the ITMA under H-C entropy power as the following:

(5) $$R_{\alpha }^{2}=1-e^{-2I_{\alpha }(T,S)}$$

where *I*_*α*_(*T*, *S*) is the mutual information between *T* and *S* under H-C entropy [32]. Specifically, for *α* ≠ 1,

(6) $$I_{\alpha }(T,S)=HC_{\alpha }(T)+HC_{\alpha }(S)+(1-\alpha )HC_{\alpha }(T)HC_{\alpha }(S)-HC_{\alpha }(T,S)$$

When *α* = 1, *I*_*α*_(*T*, *S*) = *I*(*T*, *S*) and $R_{\alpha }^{2}=R_{h}^{2}$ in Equation (1).

It is important to notice that: $\frac{EP_{\alpha }(T)-EP_{\alpha }(T|S)}{EP_{\alpha }(T)}\neq 1-e^{-2I_{\alpha }(T,S)}$, for *α* ≠ 1 where $EP_{\alpha }(X)=e^{\frac{2}{n}HC_{\alpha }(X)}/(2\pi e)$.

Some basic properties are available here.

1. When *T* and *S* are independent, *I*_*α*_(*T*, *S*) = 0. Thus, $R_{\alpha }^{2}=0$.
2. When *T* and *S* are deterministic, the value of *I*_*α*_(*T*, *S*) will depend on *α* > 1 or *α* < 1 as seem in the following propositions.

### Proposition 2.

*Let T and S be two continuously normally distributed random variables such as the joint distribution of* $(T,S{)}^{\prime }\sim N\left(\left[\begin{array}{l} \mu_{T}\\ \mu_{S} \end{array}\right],\left[\begin{array}{cc} \sigma_{T}^{2} & \rho \sigma_{T}\sigma_{S}\\ \rho \sigma_{T}\sigma_{S} & \sigma_{S}^{2} \end{array}\right]\right)$, *the conditional distribution of* $(T|S)\sim N\left(\mu_{T}+\frac{\rho \sigma_{T}}{\sigma_{S}}\left(S-\mu_{S}\right),\sigma_{T}^{2}\left(1-\rho^{2}\right)\right)$ and $T\sim N\left(\mu_{T},\sigma_{T}^{2}\right)$, *where* “′” *means vector transpose*. *Then*, *we have the following results*:

2.1 The mutual information for H-C entropy depends not only the correlation between *T* and *S*, but also their standard deviations for *α* ≠ 1.
(7) $$I_{\alpha }(T,S)=\frac{{\left(2\pi \sigma_{T}\sigma_{S}\right)}^{1-\alpha }}{\alpha (1-\alpha )}\left[1-{\left(\sqrt{1-\rho^{2}}\right)}^{1-\alpha }\right]\;\alpha \neq 1\;I_{\alpha }(T,S)=I(T,S)=-\frac{1}{2}\text{log}\left(1-\rho^{2}\right)\;\text{for}\;\alpha =1$$
2.2 When *α* ≥ 1, *ρ* → ±1, *I*_*α*_ → ∞. *ρ* → ±0, *I*_*α*_ → 0, *I*_*α*_ is an increasing function of |*ρ*|
2.3 When *α* < 1, *ρ* → ±1, *I*_*α*_ → 1. *ρ* → ±0, *I*_*α*_ → 0, *I*_*α*_ is an increasing function of |*ρ*|.

Therefore, for *α* < 1, maximum $R_{\alpha }^{2}=1-e^{-2}$. We can normalize $R_{\alpha }^{2}$ by dividing its maximum value to make normalized ${\tilde{R}}_{\alpha }^{2}$ in between 0 and 1

(8) $${\tilde{R}}_{\alpha ,\text{max}}^{2}=\frac{R_{\alpha }^{2}}{1-e^{-2}},\;\text{for}\;\alpha <1$$

### Proof.


$$HC_{\alpha }(T,S)=\int \int \frac{1}{\left(1-\alpha \right)}\left(f_{T,S}^{\alpha -1}(t,s)-1\right)f_{T,S}(t,s)\text{dtds}\;=\frac{1}{\left(1-\alpha \right)}\left[\int \int f_{T,S}^{\alpha }(t,s)\text{dtds}-1\right]\;=\frac{1}{\left(1-\alpha \right)}\left[\frac{2\pi \sigma_{T}\sigma_{S}\sqrt{1-\rho^{2}}}{\alpha {\left(2\pi \sigma_{T}\sigma_{S}\sqrt{1-\rho^{2}}\right)}^{\alpha }}-1\right]\;=\frac{1}{\left(1-\alpha \right)}\left[\frac{{\left(2\pi \sigma_{T}\sigma_{S}\sqrt{1-\rho^{2}}\right)}^{1-\alpha }}{\alpha }-1\right]$$

Similarly,

$$HC_{\alpha }(T)=\frac{1}{\left(1-\alpha \right)}\left[\frac{{\left(2\pi \sigma_{T}^{2}\right)}^{\frac{1-\alpha }{2}}}{\sqrt{\alpha }}-1\right]$$

$$HC_{\alpha }(S)=\frac{1}{\left(1-\alpha \right)}\left[\frac{{\left(2\pi \sigma_{S}^{2}\right)}^{\frac{1-\alpha }{2}}}{\sqrt{\alpha }}-1\right]$$

And

$$(1-\alpha )HC_{\alpha }(T)HC_{\alpha }(S)=\frac{1}{\left(1-\alpha \right)}\left[\frac{{\left(2\pi \sigma_{T}^{2}\right)}^{\frac{1-\alpha }{2}}}{\sqrt{\alpha }}-1\right]\left[\frac{{\left(2\pi \sigma_{S}^{2}\right)}^{\frac{1-\alpha }{2}}}{\sqrt{\alpha }}-1\right]\;=\frac{1}{\left(1-\alpha \right)}\frac{{\left(2\pi \sigma_{T}\sigma_{S}\right)}^{1-\alpha }}{\alpha }-\frac{1}{\left(1-\alpha \right)}\left[\frac{{\left(2\pi \sigma_{T}^{2}\right)}^{\frac{1-\alpha }{2}}}{\sqrt{\alpha }}+\frac{{\left(2\pi \sigma_{S}^{2}\right)}^{\frac{1-\alpha }{2}}}{\sqrt{\alpha }}\right]+\frac{1}{\left(1-\alpha \right)}$$

As such

$$I_{\alpha }(T,S)=\frac{1}{\left(1-\alpha \right)}\left[\frac{{\left(2\pi \sigma_{T}^{2}\right)}^{\frac{1-\alpha }{2}}}{\sqrt{\alpha }}-1\right]+\frac{1}{\left(1-\alpha \right)}\left[\frac{{\left(2\pi \sigma_{S}^{2}\right)}^{\frac{1-\alpha }{2}}}{\sqrt{\alpha }}-1\right]+\frac{1}{\left(1-\alpha \right)}\frac{{\left(2\pi \sigma_{T}\sigma_{S}\right)}^{1-\alpha }}{\alpha }\;-\frac{1}{\left(1-\alpha \right)}\left[\frac{{\left(2\pi \sigma_{T}^{2}\right)}^{\frac{1-\alpha }{2}}}{\sqrt{\alpha }}+\frac{{\left(2\pi \sigma_{S}^{2}\right)}^{\frac{1-\alpha }{2}}}{\sqrt{\alpha }}\right]+\frac{1}{\left(1-\alpha \right)}-\frac{1}{\left(1-\alpha \right)}\left[\frac{{\left(2\pi \sigma_{T}\sigma_{S}\sqrt{1-\rho^{2}}\right)}^{1-\alpha }}{\alpha }-1\right]\;=\frac{{\left(2\pi \sigma_{T}\sigma_{S}\right)}^{1-\alpha }}{\alpha (1-\alpha )}\left[1-{\left(\sqrt{1-\rho^{2}}\right)}^{1-\alpha }\right]$$

Finally, results in 2.2 and 2.3 can be concluded from the expression of *I*_*α*_(*T*, *S*). □

### Proposition 3.

*Let T and S be two binary outcome variables with 1 for a success and 0 for a failure such as the joint distribution of* (*T*, *S*)′ ~ *Multinomial* (*p*_0,0_, *p*_1,0_, *p*_0,1_, *p*_1,1_). *We have the following results:*

(9) $$I_{\alpha }(T,S)==\frac{1}{\left(1-\alpha \right)}\sum_{t=0}^{1}\sum_{s=0}^{1}\left\{{\left[p_{t,+}\right]}^{\alpha }{\left[p_{+,s}\right]}^{\alpha }-{\left[p_{t,s}\right]}^{\alpha }\right\}$$

3.1 *When p*_*t,s*_ = *p*_t,+_ *p*_+,*s*′_ *T and S are independent*, *I*_*α*_(*T*, *S*) = 0.
3.2 *Let* $\rho =\frac{p_{1,1}-p_{1,+}p_{+,1}}{\sqrt{p_{1,+}p_{0,+}p_{+,1}p_{+,0}}}$ *be the correlation between T and S*. If [*p*_0,0_]^*α*−1^ + [*p*_1,1_]^*α*−1^ > [*p*_1,0_]^*α*−1^ + [*p*_0,1_]^*α*−1^, *I*_*α*_(*T*, *S*) *is an increasing function of ρ for α* > 1. *For α* < 1, *I*_*α*_(*T*, *S*) *is an increasing function of ρ if* [*p*_0,0_]^*α*−1^ + [*p*_1,1_]^*α*−1^ > [*p*_1,0_]^*α*−1^ + [*p*_0,1_]^*α*−1^.
3.3 *For α* > 1, *I*_*α*_(*T*, *S*) ≤ *min*(*HC*_*α*_(*T*), *HC*_*α*_(*S*)).
3.4 *For α* < 1, *I*_*α*_(*T*, *S*) ≥ *max*(*HC*_*α*_(*T*), *HC*_*α*_(*S*)).
3.5 *For a given marginal distribution of T and S*, *there is a maximum value of mutual information as*
(10) $$I_{\alpha }(T,S)\leq HC_{\alpha }(T)+HC_{\alpha }(S)+(1-\alpha )HC_{\alpha }(T)HC_{\alpha }(S)-\text{min}\left(HC_{\alpha }(T,S)\right)=I_{\alpha ,max}.$$

*Thus*, *we can normalize ITMA as* ${\tilde{R}}_{\alpha ,max}(T,S)=\frac{R_{\alpha }(T,S)}{1-e^{-2I_{\alpha ,max}}}$.

### Proof.

Because

$$HC_{\alpha }(T,S)=\frac{1}{\left(1-\alpha \right)}\sum_{t=0}^{1}\sum_{s=0}^{1}p_{t,s}\left\{{\left[p_{t,s}\right]}^{\alpha -1}-1\right\}=\frac{1}{\left(1-\alpha \right)}\left\{\sum_{t=0}^{1}\sum_{s=0}^{1}{\left[p_{t,s}\right]}^{\alpha }-1\right\}$$

$$HC_{\alpha }(T)=\frac{1}{\left(1-\alpha \right)}\sum_{t=0}^{1}p_{t,+}\left\{{\left[p_{t,+}\right]}^{\alpha -1}-1\right\}=\frac{1}{\left(1-\alpha \right)}\left\{\sum_{t=0}^{1}{\left[p_{t,+}\right]}^{\alpha }-1\right\}$$

$$HC_{\alpha }(S)=\frac{1}{\left(1-\alpha \right)}\sum_{s=0}^{1}p_{+,s}\left\{{\left[p_{+,s}\right]}^{\alpha -1}-1\right\}=\frac{1}{\left(1-\alpha \right)}\left\{\sum_{s=0}^{1}{\left[p_{+,s}\right]}^{\alpha }-1\right\}$$

And

$$(1-\alpha )HC_{\alpha }(T)HC_{\alpha }(S)=\frac{1}{\left(1-\alpha \right)}\left\{\sum_{t=0}^{1}{\left[p_{t,+}\right]}^{\alpha }-1\right\}\left\{\sum_{s=0}^{1}{\left[p_{+,s}\right]}^{\alpha }-1\right\}$$

Thus,

$$HC_{\alpha }(T)+HC_{\alpha }(S)+(1-\alpha )HC_{\alpha }(T)HC_{\alpha }(S)=\frac{1}{\left(1-\alpha \right)}\left[\left\{\sum_{t=0}^{1}{\left[p_{t,+}\right]}^{\alpha }\right\}\left\{\sum_{s=0}^{1}{\left[p_{+,s}\right]}^{\alpha }\right\}-1\right]$$

Therefore,

$$I_{\alpha }(T,S)=\frac{1}{\left(1-\alpha \right)}\left(\left[\left\{\sum_{t=0}^{1}{\left[p_{t,+}\right]}^{\alpha }\right\}\left\{\sum_{s=0}^{1}{\left[p_{+,s}\right]}^{\alpha }\right\}-1\right]-\frac{1}{\left(1-\alpha \right)}\left\{\sum_{t=0}^{1}\sum_{s=0}^{1}{\left[p_{t,s}\right]}^{\alpha }-1\right\}\right)\;=\frac{1}{\left(1-\alpha \right)}\left[\left\{\sum_{t=0}^{1}{\left[p_{t,+}\right]}^{\alpha }\right\}\left\{\sum_{s=0}^{1}{\left[p_{+,s}\right]}^{\alpha }\right\}-\sum_{t=0}^{1}\sum_{s=0}^{1}{\left[p_{t,s}\right]}^{\alpha }\right]\;=\frac{1}{\left(1-\alpha \right)}\sum_{t=0}^{1}\sum_{s=0}^{1}\left\{{\left[p_{t,+}\right]}^{\alpha }{\left[p_{+,s}\right]}^{\alpha }-{\left[p_{t,s}\right]}^{\alpha }\right\}$$

which is the expression given in Equation (9).

Result 3.1 is the direct derivation from Equation (9) as *p*_*t,s*_ = *p*_*t*,+_*p*_+,*s*_.

Result 3.2 can be derived through the following relationship:

$$p_{1,1}=p_{1,+}p_{+,1}+\rho \sqrt{p_{1,+}p_{0,+}p_{+,1}p_{+,0}}$$

$$p_{1,0}=p_{1,+}p_{+,0}-\rho \sqrt{p_{1,+}p_{0,+}p_{+,1}p_{+,0}}$$

$$p_{0,1}=p_{0,+}p_{+,1}-\rho \sqrt{p_{1,+}p_{0,+}p_{+,1}p_{+,0}}$$

$$p_{0,0}=p_{0,+}p_{+,0}+\rho \sqrt{p_{1,+}p_{0,+}p_{+,1}p_{+,0}}$$

where

(11) $$\frac{max\left(-p_{1,+}p_{+,1},-p_{0,+}p_{+,0},p_{1,+}p_{+,0}-1,p_{0,+}p_{+,1}-1\right)}{\sqrt{p_{1,+}p_{0,+}p_{+,1}p_{+,0}}}\leq \rho \leq \frac{min\left(1-p_{1,+}p_{+,1},1-p_{0,+}p_{+,0},p_{1,+}p_{+,0},p_{0,+}p_{+,1}\right)}{\sqrt{p_{1,+}p_{0,+}p_{+,1}p_{+,0}}}$$

Then,

$$I_{\alpha }(T,S)=\frac{1}{\left(1-\alpha \right)}\sum_{t=0}^{1}\sum_{s=0}^{1}\left\{{\left[p_{t,+}\right]}^{\alpha }{\left[p_{+,s}\right]}^{\alpha }-{\left[p_{t,+}p_{+,s}+{\left(-1\right)}^{t+s}\rho \sqrt{p_{1,+}p_{0,+}p_{+,1}p_{+,0}}\right]}^{\alpha }\right\}$$

Taking the derivative of *I*_*α*_(*T*, *S*) on *ρ*, we have

$$\frac{dI_{\alpha }(T,S)}{d\rho }=\frac{\alpha }{\left(1-\alpha \right)}\sum_{t=0}^{1}\sum_{s=0}^{1}\left\{-{\left(-1\right)}^{t+s}\sqrt{p_{1,+}p_{0,+}p_{+,1}p_{+,0}}{\left[p_{t,+}^{\left(z\right)}p_{+,s}^{\left(z\right)}+{\left(-1\right)}^{t+s}\rho \sqrt{p_{1,+}p_{0,+}p_{+,1}p_{+,0}}\right]}^{\alpha -1}\right\}\;=\frac{\alpha }{\left(1-\alpha \right)}\sum_{t=0}^{1}\sum_{s=0}^{1}\sqrt{p_{1,+}p_{0,+}p_{+,1}p_{+,0}}\left\{{\left(-1\right)}^{t+s+1}{\left[p_{t,s}\right]}^{\alpha -1}\right\}\;=\frac{\alpha }{\left(1-\alpha \right)}\sqrt{p_{1,+}p_{0,+}p_{+,1}p_{+,0}}\left\{-{\left[p_{0,0}\right]}^{\alpha -1}-{\left[p_{1,1}\right]}^{\alpha -1}+{\left[p_{1,0}\right]}^{\alpha -1}+{\left[p_{0,1}\right]}^{\alpha -1}\right\}$$

Under condition that ${\left[p_{0,0}\right]}^{\alpha -1}+{\left[p_{1,1}\right]}^{\alpha -1}>{\left[p_{1,0}\right]}^{\alpha -1}+{\left[p_{0,1}\right]}^{\alpha -1}$, $\frac{dI_{\alpha }(T,S)}{d\rho }>0$ for *α* > 1. Similarly, under condition that ${\left[p_{0,0}\right]}^{\alpha -1}+{\left[p_{1,1}\right]}^{\alpha -1}<{\left[p_{1,0}\right]}^{\alpha -1}+{\left[p_{0,1}\right]}^{\alpha -1}$, $\frac{dI_{\alpha }(T,S)}{d\rho }>0$ for *α* < 1.

For 3.3 and 3.4

when *α* > 1,

$$HC_{\alpha }(T)-I_{\alpha }(T,S)=\frac{1}{\left(\alpha -1\right)}\sum_{t=0}^{1}\left\{1-{\left[p_{t,+}\right]}^{\alpha }-\sum_{s=0}^{1}\left\{{\left[p_{t,s}\right]}^{\alpha }-{\left[p_{t,+}\right]}^{\alpha }{\left[p_{+,s}\right]}^{\alpha }\right\}\right\}\;=\frac{1}{\left(\alpha -1\right)}\sum_{t=0}^{1}\left\{1-\sum_{s=0}^{1}{\left[p_{t,s}\right]}^{\alpha }-{\left[p_{t,+}\right]}^{\alpha }\left\{1-\sum_{s=0}^{1}{\left[p_{+,s}\right]}^{\alpha }\right\}\right\}\;\geq \frac{1}{\left(\alpha -1\right)}\sum_{t=0}^{1}\left\{1-\sum_{s=0}^{1}{\left[p_{+,s}\right]}^{\alpha }-{\left[p_{t,+}\right]}^{\alpha }\left\{1-\sum_{s=0}^{1}{\left[p_{+,s}\right]}^{\alpha }\right\}\right\}\;=\frac{1}{\left(\alpha -1\right)}\left\{1-\sum_{s=0}^{1}{\left[p_{+,s}\right]}^{\alpha }\right\}\sum_{t=0}^{1}\left\{1-{\left[p_{t,+}\right]}^{\alpha }\right\}\geq 0$$

However, when *α* < 1,

$$HC_{\alpha }(T)-I_{\alpha }(T,S)=\frac{1}{\left(1-\alpha \right)}\sum_{t=0}^{1}\left\{{\left[p_{t,+}\right]}^{\alpha }-1-\sum_{s=0}^{1}\left\{{\left[p_{t,+}\right]}^{\alpha }{\left[p_{+,s}\right]}^{\alpha }-{\left[p_{t,s}\right]}^{\alpha }\right\}\right\}\;=\frac{1}{\left(1-\alpha \right)}\sum_{t=0}^{1}\left\{{\left[p_{t,+}\right]}^{\alpha }-1-{\left[p_{t,+}\right]}^{\alpha }\sum_{s=0}^{1}{\left[p_{+,s}\right]}^{\alpha }+\sum_{s=0}^{1}{\left[p_{t,s}\right]}^{\alpha }\right\}\;\leq \frac{1}{\left(1-\alpha \right)}\sum_{t=0}^{1}\left\{{\left[p_{t,+}\right]}^{\alpha }-1-{\left[p_{t,+}\right]}^{\alpha }\sum_{s=0}^{1}{\left[p_{+,s}\right]}^{\alpha }+\sum_{s=0}^{1}{\left[p_{+,s}\right]}^{\alpha }\right\}\;=\frac{1}{\left(1-\alpha \right)}\sum_{t=0}^{1}\left({\left[p_{t,+}\right]}^{\alpha }-1\right)\sum_{s=0}^{1}\left(1-{\left[p_{+,s}\right]}^{\alpha }\right)\leq 0.$$

Because of a symmetric relationship between T and S, we proved results 3.4 and 3.5.

For 3.5, for fixed marginal probability, *I*_*α*_(*T*, *S*) depends only on *ρ* in *HC*_*α*_(*T*, *S*). Like 3.2,

$$\frac{dHC_{\alpha }(T,S)}{d\rho }=\frac{\alpha }{\left(1-\alpha \right)}\sqrt{p_{1,+}p_{0,+}p_{+,1}p_{+,0}}\left\{-{\left[p_{0,0}\right]}^{\alpha -1}-{\left[p_{1,1}\right]}^{\alpha -1}+{\left[p_{1,0}\right]}^{\alpha -1}+{\left[p_{0,1}\right]}^{\alpha -1}\right\}$$

When $\frac{dHC_{\alpha }(T,S)}{d\rho }>0$, taking the lower boundary of *ρ* in inequality (11) will derive the min value of *HC*_*α*_(*T*, *S*).

When $\frac{dHC_{\alpha }(T,S)}{d\rho }<0$, taking the upper boundary of *ρ* in inequality (11) will derive the min value of *HC*_*α*_(*T*, *S*). □

### Remark 1.

*When the concordant pairs are more likely than the discordant pairs for the two binary endpoints*, *for α*>1, [*p*_0,0_]^*α*−1^ + [*p*_1,1_]^*α*−1^ *is more likely to be greater than* [*p*_1,0_]^*α*−1^ + [*p*_0,1_]^*α*−1^. *However*, *when α*<1, [*p*_0,0_]^*α*−1^ + [*p*_1,1_]^*α*−1^ *is more likely to be less than* [*p*_1,0_]^*α*−1^ + [*p*_0,1_]^*α*−1^. *Thus*, *when two binary endpoints have more chance to be concordant*, *the mutual information will be an increasing function of correlation coefficient of ρ as shown in* Proposition 3 *Result 3*.*2*.

Now we define a model for a binary *T* and a continuous normally distributed surrogate variable *S*.

### Proposition 4.

*Let T be a binary outcome variable and S continuous normally distributed surrogate variable*, *where T*~*B*(*p*_0_, *p*_1_) and $S\sim N\left(\mu_{S},\sigma_{S}^{2}\right)$. *We assume that there is a latent variable U such that T* = 1 ⇔ *U* ≥ 0, i.*e*, *a Probit model with U*~*N*(*μ*_*T*_, 1) *and μ*_*T*_ = Φ^−1^(*p*_1_). *Assume a correlation coefficient between U and S* is *ρ*, *the conditional binary endpoint T*|*S follows a Bernoulli distribution with* $p_{1|s}=p_{T=1|S=s}=\Phi \left(\frac{\Phi^{-1}\left(p_{1}\right)+\rho \frac{s-\mu_{S}}{\sigma_{S}}}{\sqrt{1-\rho^{2}}}\right)$ and $p_{0|s}=p_{T=0|S=s}=1-p_{T=1|S=s}=\Phi \left(\frac{\Phi^{-1}\left(p_{0}\right)-\rho \frac{s-\mu_{S}}{\sigma_{S}}}{\sqrt{1-\rho^{2}}}\right)$. *We have the following results*:

4.1 *The mutual information for H-C entropy is*
$$I_{\alpha }(T,S)=\frac{1}{\left(1-\alpha \right)}\left\{\frac{{\left(2\pi \sigma_{S}^{2}\right)}^{\frac{1-\alpha }{2}}}{\sqrt{\alpha }}\sum_{t=0}^{1}{\left[p_{t}\right]}^{\alpha }-\int_{-\infty }^{\infty }\left[\sum_{t=0}^{1}{\left[p_{t|s}\right]}^{\alpha }\right]\phi^{\alpha }\left(\frac{s-\mu_{S}}{\sigma_{S}}\right)ds\right\}\;=\frac{{\left(2\pi \sigma_{S}^{2}\right)}^{\frac{1-\alpha }{2}}}{(1-\alpha )\sqrt{\alpha }}\sum_{t=0}^{1}\left[{\left(p_{t}\right)}^{\alpha }-{ℑ}^{\left(\alpha \right)}\left(\frac{\Phi^{-1}\left(p_{t}\right)}{\sqrt{1-\rho^{2}}},\frac{{\left(-1\right)}^{1-t}\frac{\rho }{\sqrt{\alpha }}}{\sqrt{1-\rho^{2}}}\right)\right]$$
  *where* ${ℑ}^{\left(\alpha \right)}(a,b)=\int_{-\infty }^{\infty }\Phi^{\alpha }(a+by)\phi (y)dy$.
4.2 *When ρ* = 0, *I*_*α*_(*T*, *S*) = 0.
4.3 *When ρ* → ±1, $I_{\alpha }(T,S)\to \frac{1}{\left(1-\alpha \right)}\frac{{\left(2\pi \sigma_{S}^{2}\right)}^{\frac{1-\alpha }{2}}}{\sqrt{\alpha }}\left\{\sum_{t=0}^{1}{\left[p_{t}\right]}^{\alpha }-\Phi \left(\sqrt{\alpha }\Phi^{-1}\left(p_{1}\right)\right)-\Phi \left(\sqrt{\alpha }\Phi^{-1}\left(p_{0}\right)\right)\right\}$
4.4 *For α* → 1, $I_{\alpha }(T,S)\to \sum_{t=0}^{1}\left[\int_{-\infty }^{\infty }p_{t|s}log\left(p_{t|s}\right)\phi (s)ds-p_{t}log\left(p_{t}\right)\right]]$

### Proof.

The joint distribution function for (*T*, *S*) = (*t*, *s*) is:

$$f(t,s)=\;{\left\{\int_{0}^{\infty }\frac{1}{2\pi \sigma_{S}\sqrt{1-\rho^{2}}}exp\left(-\frac{1}{2}\left[\begin{array}{l} \begin{array}{l} \begin{array}{ll} u-\mu_{T} & \frac{S-\mu_{S}}{\sigma_{S}} \end{array} \end{array} \end{array}\right]{\left[\begin{array}{ll} 1 & \rho \\ \rho & 1 \end{array}\right]}^{-1}\left[\begin{array}{c} u-\mu_{T}\\ \frac{S-\mu_{S}}{\sigma_{S}} \end{array}\right]\right)du\right\}}^{t}\;\times {\left\{\int_{-\infty }^{0}\frac{1}{2\pi \sigma_{S}\sqrt{1-\rho^{2}}}exp\left(-\frac{1}{2}\left[\begin{array}{l} \begin{array}{l} \begin{array}{ll} u-\mu_{T} & \frac{S-\mu_{S}}{\sigma_{S}} \end{array} \end{array} \end{array}\right]{\left[\begin{array}{ll} 1 & \rho \sigma_{S}\\ \rho \sigma_{S} & \sigma_{S}^{2} \end{array}\right]}^{-1}\left[\begin{array}{c} u-\mu_{T}\\ \frac{S-\mu_{S}}{\sigma_{S}} \end{array}\right]\right)du\right\}}^{1-t}\;={\left\{\int_{0}^{\infty }\frac{1}{2\pi \sigma_{S}\sqrt{1-\rho^{2}}}exp\left(-\frac{1}{2\left(1-\rho^{2}\right)}\left({\left[u-\mu_{T}-\rho \frac{S-\mu_{S}}{\sigma_{S}}\right]}^{2}+\left(1-\rho^{2}\right){\left[\frac{S-\mu_{S}}{\sigma_{S}}\right]}^{2}\right)\right)du\right\}}^{t}\;\times {\left\{\int_{-\infty }^{0}\frac{1}{2\pi \sigma_{S}\sqrt{1-\rho^{2}}}exp\left(-\frac{1}{2\left(1-\rho^{2}\right)}\left({\left[u-\mu_{T}-\rho \frac{S-\mu_{S}}{\sigma_{S}}\right]}^{2}+\left(1-\rho^{2}\right){\left[\frac{S-\mu_{S}}{\sigma_{S}}\right]}^{2}\right)\right)du\right\}}^{1-t}\;={\left[1-\Phi \left(\frac{-\mu_{T}-\rho \frac{S-\mu_{S}}{\sigma_{S}}}{\sqrt{1-\rho^{2}}}\right)\right]}^{t}{\left[\Phi \left(\frac{-\mu_{T}-\rho \frac{S-\mu_{S}}{\sigma_{S}}}{\sqrt{1-\rho^{2}}}\right)\right]}^{1-t}\frac{\phi \left(\frac{S-\mu_{S}}{\sigma_{S}}\right)}{\sigma_{S}}\;={\left[1-\Phi \left(\frac{-\Phi^{-1}\left(p_{1}\right)-\rho \frac{S-\mu_{S}}{\sigma_{S}}}{\sqrt{1-\rho^{2}}}\right)\right]}^{t}{\left[\Phi \left(\frac{-\Phi^{-1}\left(p_{1}\right)-\rho \frac{S-\mu_{S}}{\sigma_{S}}}{\sqrt{1-\rho^{2}}}\right)\right]}^{1-t}\frac{\phi \left(\frac{S-\mu_{S}}{\sigma_{S}}\right)}{\sigma_{S}}\;={\left[p_{1|s}\right]}^{t}{\left[p_{0|s}\right]}^{1-t}\frac{\phi \left(\frac{s-\mu_{S}}{\sigma_{S}}\right)}{\sigma_{S}}$$

Therefore,

$$HC_{\alpha }(T)=\frac{1}{\left(1-\alpha \right)}\left\{\sum_{t=0}^{1}{\left[p_{t}\right]}^{\alpha }-1\right\}\;HC_{\alpha }(S)=\frac{1}{\left(1-\alpha \right)}\left[\frac{{\left(2\pi \sigma_{S}^{2}\right)}^{\frac{1-\alpha }{2}}}{\sqrt{\alpha }}-1\right]\;HC_{\alpha }(T,S)=\frac{1}{1-\alpha }\left\{\sigma_{S}^{1-\alpha }\int_{-\infty }^{\infty }\left[p_{1|s}^{\alpha }+p_{0|s}^{\alpha }\right]\phi^{\alpha }\left(\frac{S-\mu_{S}}{\sigma_{S}}\right)d\frac{S}{\sigma_{S}}-1\right\}\;HC_{\alpha }(T)+HC_{\alpha }(S)+(1-\alpha )HC_{\alpha }(T)HC_{\alpha }(S)=\frac{1}{\left(1-\alpha \right)}\left\{\frac{{\left(2\pi \sigma_{S}^{2}\right)}^{\frac{1-\alpha }{2}}}{\sqrt{\alpha }}\sum_{t=0}^{1}{\left[p_{t}\right]}^{\alpha }-1\right\}\;I_{\alpha }(T,S)\;=HC_{\alpha }(T)+HC_{\alpha }(S)+(1-\alpha )HC_{\alpha }(T)HC_{\alpha }(S)-HC_{\alpha }(T,S)\;=\frac{1}{\left(1-\alpha \right)}\left\{\frac{{\left(2\pi \sigma_{S}^{2}\right)}^{\frac{1-\alpha }{2}}}{\sqrt{\alpha }}\sum_{t=0}^{1}{\left[p_{t}\right]}^{\alpha }-\int_{-\infty }^{\infty }\left[\sum_{t=0}^{1}p_{t|s}\alpha \right]\frac{\phi^{\alpha }\left(\frac{s-\mu_{S}}{\sigma_{S}}\right)}{\sigma_{S}^{\alpha }}ds\right\}$$

Therefore,

$$HC_{\alpha }(T)=\frac{1}{\left(1-\alpha \right)}\left\{\sum_{t=0}^{1}{\left[p_{t}\right]}^{\alpha }-1\right\}\;HC_{\alpha }(S)=\frac{1}{\left(1-\alpha \right)}\left[\frac{{\left(2\pi \sigma_{S}^{2}\right)}^{\frac{1-\alpha }{2}}}{\sqrt{\alpha }}-1\right]\;HC_{\alpha }(T,S)=\frac{1}{1-\alpha }\left\{{\sigma_{S}}^{1-\alpha }\int_{-\infty }^{\infty }\left[{p_{1|s}}^{\alpha }+{p_{0|s}}^{\alpha }\right]\phi^{\alpha }\left(\frac{S-\mu_{S}}{\sigma_{S}}\right)d\frac{S}{\sigma_{S}}-1\right\}\;HC_{\alpha }(T)+HC_{\alpha }(S)+(1-\alpha )HC_{\alpha }(T)HC_{\alpha }(S)=\frac{1}{\left(1-\alpha \right)}\left\{\frac{{\left(2\pi \sigma_{S}^{2}\right)}^{\frac{1-\alpha }{2}}}{\sqrt{\alpha }}\sum_{t=0}^{1}{\left[p_{t}\right]}^{\alpha }-1\right\}\;I_{\alpha }(T,S)\;=HC_{\alpha }(T)+HC_{\alpha }(S)+(1-\alpha )HC_{\alpha }(T)HC_{\alpha }(S)-HC_{\alpha }(T,S)\;=\frac{1}{\left(1-\alpha \right)}\left\{\frac{{\left(2\pi \sigma_{S}^{2}\right)}^{\frac{1-\alpha }{2}}}{\sqrt{\alpha }}\sum_{t=0}^{1}{\left[p_{t}\right]}^{\alpha }-\int_{-\infty }^{\infty }\left[\sum_{t=0}^{1}{p_{t|s}}^{\alpha }\right]\frac{\phi^{\alpha }\left(\frac{s-\mu_{S}}{\sigma_{S}}\right)}{{\sigma_{S}}^{\alpha }}ds\right\}$$

Using $J^{\left(\alpha \right)}(a,b)=\int_{-\infty }^{\infty }\Phi^{\alpha }(a+by)\phi (y)dy$, we can derive an alternative formulation

$$\int_{-\infty }^{\infty }\left[\sum_{t=0}^{1}\Phi {\left(\frac{\Phi^{-1}\left(p_{t}\right)+{\left(-1\right)}^{1-t}\rho \frac{s-\mu_{S}}{\sigma_{S}}}{\sqrt{1-\rho^{2}}}\right)}^{\alpha }\right]\frac{\phi^{\alpha \left(\frac{s-\mu_{S}}{\sigma_{S}}\right)}}{{\sigma_{S}}^{\alpha }}ds\;=\int_{-\infty }^{\infty }\left[\sum_{t=0}^{1}\Phi {\left(\frac{\Phi^{-1}\left(p_{t}\right)+{\left(-1\right)}^{1-t}\rho \frac{S-\mu_{S}}{\sigma_{S}}}{\sqrt{1-\rho^{2}}}\right)}^{\alpha }\right]\frac{e^{-\frac{\alpha }{2\sigma_{S}^{2}{\left(s-\mu_{S}\right)}^{2}}}}{{\left(2\pi \sigma_{S}^{2}\right)}^{\frac{\alpha }{2}}}ds\;=\frac{{\left(2\pi \sigma_{S}^{2}/\alpha \right)}^{\frac{1}{2}}}{{\left(2\pi \sigma_{S}^{2}\right)}^{\frac{\alpha }{2}}}\int_{-\infty }^{\infty }\left[\sum_{t=0}^{1}\Phi {\left(\frac{\Phi^{-1}\left(p_{t}\right)+{\left(-1\right)}^{1-t}\frac{\rho }{\sqrt{\alpha }}y}{\sqrt{1-\rho^{2}}}\right)}^{\alpha }\right]\frac{e^{-\frac{1}{2}y^{2}}}{{\left(2\pi \right)}^{\frac{1}{2}}}dy\;=\frac{{\left(2\pi \sigma_{S}^{2}\right)}^{\frac{1-\alpha }{2}}}{\sqrt{\alpha }}\sum_{t=0}^{1}\int_{-\infty }^{\infty }\Phi {\left(\frac{\Phi^{-1}\left(p_{t}\right)+{\left(-1\right)}^{1-t}\frac{\rho }{\sqrt{\alpha }}y}{\sqrt{1-\rho^{2}}}\right)}^{\alpha }\phi (y)dy$$

*I*_*α*_(*T*, *S*)can be simplified as

$$I_{\alpha }(T,S)=\frac{{\left(2\pi \sigma_{S}^{2}\right)}^{\frac{1-\alpha }{2}}}{(1-\alpha )\sqrt{\alpha }}\sum_{t=0}^{1}\left[{\left(p_{t}\right)}^{\alpha }-{ℑ}^{\left(\alpha \right)}\left(\frac{\Phi^{-1}\left(p_{t}\right)}{\sqrt{1-\rho^{2}}},\frac{{\left(-1\right)}^{1-t}\frac{\rho }{\sqrt{\alpha }}}{\sqrt{1-\rho^{2}}}\right)\right]$$

Thus, we complete the proof for 4.1.

For 4.2, *ρ* = 0,

$$I_{\alpha }(T,S)=\frac{1}{\left(1-\alpha \right)}\left\{\frac{{\left(2\pi \sigma_{S}^{2}\right)}^{\frac{1-\alpha }{2}}}{\sqrt{\alpha }}\sum_{t=0}^{1}{\left[p_{t}\right]}^{\alpha }-\int_{-\infty }^{\infty }\left[{p_{1}}^{\alpha }+{p_{0}}^{\alpha }\right]\frac{\phi^{\alpha }\left(\frac{S-\mu_{S}}{\sigma_{S}}\right)}{{\sigma_{S}}^{\alpha }}ds\right\}=0$$

For 4.3, as *ρ* → 1,

$$\int_{-\infty }^{\infty }\left[{p_{1|s}}^{\alpha }+{p_{0|s}}^{\alpha }\right]\frac{\phi^{\alpha }\left(\frac{s-\mu_{S}}{\sigma_{S}}\right)}{{\sigma_{S}}^{\alpha }}ds\to \int_{-\Phi^{-1}\left(p_{1}\right)}^{\infty }\frac{\phi^{\alpha }\left(\frac{s-\mu_{S}}{\sigma_{S}}\right)}{{\sigma_{S}}^{\alpha }}ds+\int_{-\infty }^{\Phi^{-1}\left(p_{0}\right)}\frac{\phi^{\alpha }\left(\frac{s-\mu_{S}}{\sigma_{S}}\right)}{{\sigma_{S}}^{\alpha }}ds\;=\frac{{\left(2\pi \sigma_{S}^{2}\right)}^{\frac{1-\alpha }{2}}}{\sqrt{\alpha }}\left[\Phi \left(\sqrt{\alpha }\Phi^{-1}\left(p_{1}\right)\right)+\Phi \left(\sqrt{\alpha }\Phi^{-1}\left(p_{0}\right)\right)\right]$$

Similarly, *ρ* → −1, $\int_{-\infty }^{\infty }\left[{p_{1|s}}^{\alpha }+{p_{0|s}}^{\alpha }\right]\frac{\phi^{\alpha }\left(\frac{s-\mu_{S}}{\sigma_{S}}\right)}{{\sigma_{S}}^{\alpha }}ds\to \frac{{\left(2\pi \sigma_{S}^{2}\right)}^{\frac{1-\alpha }{2}}}{\sqrt{\alpha }}\left[\Phi \left(\sqrt{\alpha }\Phi^{-1}\left(p_{1}\right)\right)+\Phi \left(\sqrt{\alpha }\Phi^{-1}\left(p_{0}\right)\right)\right]$.

So, *ρ* → ±1, $I_{\alpha }(T,S)\to \frac{1}{\left(1-\alpha \right)}\frac{{\left(2\pi \sigma_{S}^{2}\right)}^{\frac{1-\alpha }{2}}}{\sqrt{\alpha }}\left\{\sum_{t=0}^{1}\left[{\left(p_{t}\right)}^{\alpha }-\Phi \left(\sqrt{\alpha }\Phi^{-1}\left(p_{t}\right)\right)\right]\right\}$.

For *α* = 1, H-C entropy is similar to Shannon’s entropy. Thus, by taking limit of *α* to 1, we can derive Shannon’s mutual information for the Probit model in 4.4.


$${\text{lim}}_{\alpha \to 1}I_{\alpha }(T,S)={\text{lim}}_{\alpha \to 1}\left\{\frac{{\left(2\pi \sigma_{S}^{2}\right)}^{\frac{1-\alpha }{2}}}{(1-\alpha )\sqrt{\alpha }}\sum_{t=0}^{1}\left[{\left(p_{t}\right)}^{\alpha }-\int_{-\infty }^{\infty }\Phi {\left(\frac{\Phi^{-1}\left(p_{t}\right)+{\left(-1\right)}^{1-t}\frac{\rho }{\sqrt{\alpha }}y}{\sqrt{1-\rho^{2}}}\right)}^{\alpha }\phi (y)dy\right]\right\}\;={\text{lim}}_{\alpha \to 1}\left\{\frac{1}{\left(1-\alpha \right)}\sum_{t=0}^{1}\left[{\left(p_{t}\right)}^{\alpha }-\int_{-\infty }^{\infty }\Phi {\left(\frac{\Phi^{-1}\left(p_{t}\right)+{\left(-1\right)}^{1-t}\frac{\rho }{\sqrt{\alpha }}y}{\sqrt{1-\rho^{2}}}\right)}^{\alpha }\phi (y)dy\right]\right\}\;={\text{lim}}_{\alpha \to 1}\left\{\frac{1}{-1}\sum_{t=0}^{1}\left[{\left(p_{t}\right)}^{\alpha }log\left(p_{t}\right)-\int_{-\infty }^{\infty }\Phi {\left(\frac{\Phi^{-1}\left(p_{t}\right)+{\left(-1\right)}^{1-t}\frac{\rho }{\sqrt{\alpha }}y}{\sqrt{1-\rho^{2}}}\right)}^{\alpha }log\left(\Phi \left(\frac{\Phi^{-1}\left(p_{t}\right)+{\left(-1\right)}^{1-t}\frac{\rho }{\sqrt{\alpha }}y}{\sqrt{1-\rho^{2}}}\right)\right)\phi (y)dy\right]\right\}\;=\sum_{t=0}^{1}\left[\int_{-\infty }^{\infty }\Phi \left(\frac{\Phi^{-1}\left(p_{t}\right)+{\left(-1\right)}^{1-t}\rho y}{\sqrt{1-\rho^{2}}}\right)log\left(\Phi \left(\frac{\Phi^{-1}\left(p_{t}\right)+{\left(-1\right)}^{1-t}\rho y}{\sqrt{1-\rho^{2}}}\right)\right)\phi (y)dy-p_{t}log\left(p_{t}\right)\right]=\sum_{t=0}^{1}\left[\int_{-\infty }^{\infty }p_{t|y}log\left(p_{t|y}\right)\phi (y)dy-p_{t}log\left(p_{t}\right)\right]$$

### Remark 2.

*Taking into account that* $J^{\left(2\right)}(a,b)=\Phi \left(\frac{a}{\sqrt{1+b^{2}}}\right)-2T\left(\frac{a}{\sqrt{1+b^{2}}},\frac{1}{\sqrt{1+2b^{2}}}\right)$, *where* $T(h,k)=\phi (h)\int_{0}^{k}\frac{\phi (hx)}{1+x^{2}}dx$ *is the Owen’s function* [33] *and the property T*(*h*, *k*) = *T*(−*h*, *k*), *we can derive explicit formula for α*=2,

$$I_{2}(T,S)=-\frac{1}{2\sqrt{\pi }\sigma_{S}}\sum_{t=0}^{1}\left[{\left(p_{t}\right)}^{2}-\left[\Phi \left(\frac{\Phi^{-1}\left(p_{t}\right)}{\sqrt{1-\rho^{2}/2}}\right)-2T\left(\frac{\Phi^{-1}\left(p_{t}\right)}{\sqrt{1-\rho^{2}/2}},\sqrt{\left(1-\rho^{2}\right)}\right)\right]\right]$$

*Since* Φ^−1^(*p*_*t*_) = Φ^−1^(1 – *p*_1−*t*_) = −Φ^−1^(*p*_1−*t*_) and $\Phi \left(\frac{\Phi^{-1}\left(p_{1}\right)}{\sqrt{1-\rho^{2}/2}}\right)+\Phi \left(\frac{\Phi^{-1}\left(p_{0}\right)}{\sqrt{1-\rho^{2}/2}}\right)=1$ *we can simplify the expression as*

$$I_{2}(T,S)=\frac{1}{2\sqrt{\pi }\sigma_{S}}\left\{1-4T\left(\frac{\Phi^{-1}\left(p_{0}\right)}{\sqrt{1-\rho^{2}/2}},\sqrt{\left(1-\rho^{2}\right)}\right)-{\left(p_{1}\right)}^{2}-{\left(p_{0}\right)}^{2}\right\}$$

## 3. Surrogacy of a Longitudinal Biomarker for a Binary Clinical Endpoint

### 3.1. Model for Longitudinal Continuous Surrogate Biomarkers in Phase II Trials

In many phase II trials, clinical endpoints of interest (*T*) are often a proportion of a binary endpoint or mean of a continuous variable. For example, in oncology phase II trials, a common clinical endpoint is total response rate. The surrogate biomarkers, on the other hand, are usually lab tests either from serum or urine or imaging modalities that can be measured repeatedly during the study. In this section, we focus on a binary one-time clinical endpoint *T* and a continuous repeated surrogate variable *S*.

In the remainder of the paper, we will use *t*_*j*_ to denote the time of *j*th measurement, since baseline in a longitudinal trial. For simplicity, consider the difference model from baseline *t*_0_ = 0.

Let the general model as:

$$S_{i,j}|Z_{i},T_{i}=\mu_{S_{i}}+\alpha_{1}Z_{i}+\alpha_{2}t_{j}+\alpha_{3}Z_{i}t_{j}+\beta_{j}T_{i}+\epsilon_{S,ij},\;\text{for}\;j=1,\ldots ,K.$$

Thus, $S_{i}|Z_{i},T_{i}={\left(S_{i,0}\left|Z_{i},T_{i},S_{i,1}\right|Z_{i},T_{i},\ldots ,S_{i,K}|Z_{i},T_{i}\right)}^{\prime }\sim MVN\left(\mu_{S_{i}},\Sigma_{SS}\right)$, where

$$\mu_{S_{i}}={\left(\mu_{S}+\alpha_{1}Z_{i}+\left(\alpha_{2}+\alpha_{3}Z_{i}\right)t_{1}+\beta_{1}T_{i},\ldots ,\mu_{S}+\alpha_{1}Z_{i}+\left(\alpha_{2}+\alpha_{3}Z_{i}\right)t_{K}+\beta_{K}T_{i}\right)}^{\prime }$$

Using a bivariate probit model [34] for the joint distribution of (*Z*_*i*_, *T*_*i*_)′ we can derive a probit model for the conditional joint distribution for (*Z*_*i*_, *T*_*i*_)′|*S*:

$$\left[\begin{array}{l} \begin{array}{l} \Phi^{-1}\left[P\left(T_{i}=1\right)\right]\\ \Phi^{-1}\left[P\left(Z_{i}=1\right)\right] \end{array}|S_{i} \end{array}\right]\sim MVN_{2}\left(\left[\begin{array}{ll} \mu_{0,1} & \gamma_{1}^{\prime }\\ \mu_{0,2} & \gamma_{2}^{\prime } \end{array}\right]\left[\begin{array}{l} 1\\ S_{i} \end{array}\right],\left[\begin{array}{ll} 1 & \rho \\ \rho & 1 \end{array}\right]\right)$$

where *μ*_0,*k*_ and *γ*_*k*_ are the intercept and regression coefficient vector for the probit regression for *T* given longitudinal *S* (*k* = 1) and for *Z* given longitudinal *S* (*k* = 2), respectively, and *ρ* is the correlation coefficient of two underlying latent normal variables for *T* and *Z*.

Because a linear combination of multivariate normal variables is still a normal random variable, we can use the Proposition 4 to calculate ITMA under H-C entropy power to evaluate surrogacy of the longitudinal biomarker in each arm conditioning on *Z*. We can also average over the treatment arms to get the mean trial level ITMA under H-C entropy, denoted as

$$I_{\alpha }(T,S|Z)=E\left[I_{\alpha }(T,S|Z)\right]=I_{\alpha }(T,S|Z=1)P(Z=1)+I_{\alpha }(T,S|Z=0)P(Z=0)$$

Furthermore, we can use the mutual information *I*_*α*_(*T*, *Z*|*S*) to verify Prentice’s criteria as suggested by [24]: i.e, conditioning on surrogate *S*, the clinical endpoint *T* and treatment assignment *Z* are independent, thus a good surrogate should lead to *I*_*α*_(*T*, *Z*|*S*) ≈ 0. Since

$$I_{\alpha }(T,Z|S)=E\langle HC_{\alpha }(T|S)+HC_{\alpha }(Z|S)+(1-\alpha )HC_{\alpha }(T|S)HC_{\alpha }(Z|S)-HC_{\alpha }(T,Z|S)\rangle$$

where

(12) $$HC_{\alpha }(T|S)=\frac{1}{1-\alpha }\left\{{\left[p(T=1|S)\right]}^{\alpha }+{\left[p(T=0|S)\right]}^{\alpha }-1\right\}$$

$$HC_{\alpha }(Z|S)=\frac{1}{1-\alpha }\left\{{\left[p(Z=1|S)\right]}^{\alpha }+{\left[p(Z=0|S)\right]}^{\alpha }-1\right\}$$

$$HC_{\alpha }(T,Z|S)=\frac{1}{1-\alpha }\left\{{\left[p(T=1,Z=1|S)\right]}^{\alpha }+{\left[p(T=1,Z=0|S)\right]}^{\alpha }+{\left[p(T=0,Z=1|S)\right]}^{\alpha }+{\left[p(T=0,Z=0|S)\right]}^{\alpha }-1\right\}$$

So for *α* ≠ 1, $I_{\alpha }(T,Z|S)=\frac{1}{1-\alpha }E\langle \sum_{t=0}^{1}\sum_{z=0}^{1}\left\{{\left[p(T=t|S)\right]}^{\alpha }{\left[p(Z=z|S)\right]}^{\alpha }-{\left[p\left(T=t,Z=z|S\right)\right]}^{\alpha }\right\}\rangle$ When *α* = 1, $I_{\alpha }(T,Z|S)=E\langle -\sum_{t=0}^{1}p(T=t|S)log[p(T=t|S)]-\sum_{z=0}^{1}p(Z=z|S)log[p(Z=z|S)]+\sum_{t=0}^{1}\sum_{z=0}^{1}p(T=t,Z=z|S)log[p(T=t,Z=z|S)]\rangle$.

For real data, we can use bivariate probit model to estimate equations for [*p*(*T* = *t*|*S*)]^*α*^, [*p*(*Z* = *z*|*S*)]^*α*^, and [*p*(*T* = *t*, *Z* = *z*|*S*)]^*α*^, then use Equation (9) to perform numerical integration for the derivation of *I*_*α*_(*T*, *Z*|*S*). One way to perform this analysis is to use R-package mvProbit from CRAN-R (https://cran.r-project.org/web/packages/mvProbit/mvProbit.pdf, accessible on January 29, 2022).

### 3.2. A Data Example

“Safety, Tolerability and Activity Study of Ibudilast in Subjects with Progressive Multiple Sclerosis” (NCT01982942) is a US National Institute of Health (NIH) sponsored multicenter, randomized, double-blind, placebo-controlled, parallel-group phase II study from November 2013 to December 2017. The main study results have been published by Fox, et al. [35]. The trial data is publicly available upon request to NIH. We use this data for the numerical illustration for H-C ITMA.

More specifically, patients were enrolled with primary or secondary progressive multiple sclerosis of this phase II randomized trial of oral ibudilast (≤100 mg daily) or placebo for 96 weeks. The primary efficacy end point was the rate of brain atrophy, as measured by the brain parenchymal fraction (brain size relative to the volume of the outer surface contour of the brain). Major secondary end points included the change in the pyramidal tracts on diffusion tensor imaging and cortical atrophy, all measures of tissue damage in multiple sclerosis.

We requested and received data from the study team that contained 104 placebo patients and 99 treated patients, with longitudinal observations in brain parenchymal fraction (BPF) and thinning of the cortical gray matter (cortical thickness) measured by magnetic resonance imaging at week 0, 24, 48, 72, and 96. For illustration purposes, we altered the primary and secondary endpoints of the trial and created a binary clinical endpoint as the cortical thickness (CTH) greater than 3 mm as a clinical outcome for less cortical gray matter atrophy and used BPF as the continuous longitudinal marker. Table 1 provides a summary of the data used for this illustration.

From Table 1, we can see that 104 patients were randomized to the control arm and 99 patients to the treatment arm. The treatment significantly reduced cortical atrophy for 71% patients who maintained more than 3 mm cortical thickness (CTH) in the treatment arm in comparison to 48% in the placebo arm at 96 weeks post baseline. While the differences in BPFs between treatment arms had p-values above 0.38 in each follow-up MRI, the aggregated changes over time measured by the slopes of a mixed random effects regression model achieved highly statistical significance with a p-value of 0.0056.

The importance of evaluating the surrogacy of the longitudinal BPF measurements for the binary CTH endpoint in MS trials is to understand the strength of surrogacy and whether it can be used to shorten trial duration. More importantly for future trial design, we need to understand how often and when the longitudinal measurements should be performed.

Using formulas derived in Proposition 4, we derived the mean mutual information and ITMA of longitudinal BPF as a surrogate for the clinical endpoint of maintaining more than 3 mm cortical thickness at the end of 96 weeks. We explore three choices of *α* = 0.5, 1, and 2 to show the difference between H-C and Shannon entropies. The value of *α* = 1 has been considered because it corresponds to Shannon entropy. The other two alpha values have been considered in other papers such as [32]. The columns of Table 2 are organized according to values of *α*. Each row in Table 2 represents a design to use BPF in the baseline (week 0) and different follow-up visits to construct a longitudinal surrogate endpoint. For example, the first row used the baseline and week 24 data while the last row used the data from baseline, weeks 48 and 72.

From Table 2, we can see that the longitudinal BPF measures at the baseline with at least one follow-up visit were all reasonable surrogates for the binary endpoint of CTH > 3.0 mm. H-C entropy with *α* = 0.5 was not sensitive enough to differentiate subtle differences in surrogacy utility of different designs to collect surrogate endpoints. When *α* = 1, H-C entropy is Shannon entropy and it was able to discriminate among different designs to the 3rd decimal place. H-C entropy for *α* = 2 was more sensitive and showed differences in all designs. As it demonstrated, using longitudinal BPF data could shorten trial duration to 72 weeks. For a trial ended at 72 weeks, additional BPF measures at week 24 and week 48 did not add any more valuable utility to surrogacy than a single measure in week 24. Overall, the p-values from the linear mixed random effects model reflected the directions of ITMAs, but not in completely concordance, perhaps, due to random variation in fitting the mixed random effects and the probit models.

Table 3 examines the longitudinal surrogacy based on Prentice’s criteria. Here we want to determine if *I*_*α*_(*T*, *Z*|*S*) is close to 0. The results of Table 3 confirm the observations in Table 2 that the longitudinal BPF is a good surrogate variable for binary CTH > 3.0 mm at 96 weeks. Because Table 3 uses the same model as Table 2, the p-values for longitudinal models are omitted. Once again, *I*_*α*_(*T*, *Z*|*S*) decreases with *α*.

## 4. Conclusions

Alonso et al. [24] proposed to assess the validity of a surrogate endpoint in terms of uncertainty reduction. The main proposals for measures of uncertainty are found in information theory. These authors based their proposal in the well-known Shannon entropy. In the past there has been an extensive work on generalized entropies [30–32,36–39]. We focus on the Havrda-Charvat entropy, which reduces to the Shannon case if the parameter is set to one, to extend that surrogacy measure. Based on the generalized entropy, we consider a generalized mutual information as it has been proved in other contexts to have better performance of some members of this family [30–32]. In this paper, the theoretical development of these measures has been completed. The advantage of our proposal is that it contains a particular case of a useful measure to assess surrogacy and demonstrates the ability to easily explore other measures which may have performance advantages for specific questions. We have seen the advantage of using *α* = 2 instead of *α* = 1 in our example to evaluate scheduling of longitudinal visits.

Some additional issues are pending. On one hand, we are working to carry out a more extensive numerical study for assessing the performance of these measures in the endpoint surrogacy context. In our paper, we compared the performance of ITMA in a real trial with three choices of *α* (0.5, 1 and 2). They were chosen for illustration purposes. The optimal choice of *α* remains a research question. Additional research can consider other ITMA, such as divergence measures [36], taking into account that the mutual information is equal to the Kullback divergence, or measures of unilateral dependency as that defined by Andonie et al. [37] based on the informational energy [39] or surrogacy for testing of variances [38].

## Figures and Tables

**Table 1.** Summary Statistics for The Real Data Example.

Variable	Control (N = 104)	Treatment (N = 99)	*p*-Value *
CTH > 3 mm: N (%)	50 (48%)	70 (71%)	0.0016
BPF: Mean (SD)			
Week 0	0.8023 (0.0301)	0.8040 (0.0281)	0.6823
Week 24	0.8012 (0.0301)	0.8039 (0.0277)	0.5001
Week 48	0.8009 (0.0311)	0.8036 (0.0282)	0.5115
Week 72	0.8001 (0.0303)	0.8032 (0.0283)	0.4433
Week 96	0.7989 (0.0306)	0.8026 (0.0293)	0.3813
Change/24 weeks **	−0.0008 (0.0001)	−0.0004 (0.0001)	0.0056

* *p*-value for CTH > 3 mm was calculated using Fisher’s exact test; *p*-values for mean differences at follow-up visits were calculated using a *t*-test. P-value for changes in 24 weeks (slopes) was calculated by the mixed random effects model.

** change per 24 weeks was estimated using a mixed random effects linear regression model using the R-lmer package.

**Table 2.** H-C Mutual Information and ITMA by Different Longitudinal Designs.

BPF Data Used	*α* = 0. 5		*α* = 1		*α* = 2		*p*-Value *
	*I*_*α*_(*T*, *S*|*Z*)	ITMA	*I*_*α*_(*T*, *S*|*Z*)	ITMA	*I*_*α*_(*T*, *S*|*Z*)	ITMA	
0, 24	4.6042	0.9999	2.6300	0.9948	0.6063	0.7026	0.0797
0, 24, 48	4.6117	0.9999	2.6307	0.9948	0.6066	0.7028	0.1025
0, 24, 48, 72	4.6209	0.9999	2.6352	0.9949	0.6071	0.7031	0.0390
0, 24, 48, 72, 96	4.6103	0.9999	2.6361	0.9949	0.6069	0.7029	0.0056
0, 48	4.4683	0.9999	2.6012	0.9945	0.5980	0.6976	0.1586
0, 72	4.4522	0.9999	2.5912	0.9944	0.5962	0.6965	0.0675
0, 24, 72	4.6223	0.9999	2.6348	0.9949	0.6072	0.7031	0.0485
0, 48, 72	4.4696	0.9999	2.6022	0.9945	0.5980	0.6976	0.0382

* *p*-value for treatment and visit interactions in a linear mixed random effects model using the R-lmer function.

**Table 3.** Prentice Criteria for Surrogate Endpoint.

BPF Data Used	*α* = 0. 5		*α* = 1		*α* = 2	
	*I*_*α*_(*T*, *Z*|*S*)	ITMA	*I*_*α*_(*T*, *Z*|*S*)	ITMA	*I*_*α*_(*T*, *Z*|*S*)	ITMA
0, 24	0.0390	0.0751	0.0271	0.0528	0.0108	0.0213
0, 24, 48	0.0388	0.0747	0.0270	0.0526	0.0108	0.0213
0, 24, 48, 72	0.0407	0.0782	0.0280	0.0545	0.0110	0.0218
0, 24, 48, 72, 96	0.0395	0.0760	0.0274	0.0533	0.0110	0.0218
0, 48	0.0428	0.0820	0.0297	0.0578	0.0117	0.0231
0, 72	0.0416	0.0798	0.0287	0.0558	0.0111	0.0219
0, 24, 72	0.0403	0.0775	0.0278	0.0541	0.0110	0.0217
0, 48, 72	0.0434	0.0832	0.0299	0.0580	0.0116	0.0229

## Data Availability

Data can be requested through the US National Institute of Neurological Disorders and Stroke and Fox, the Principal Investigator of NCT01982942 for access the de-identified data from the “Safety, Tolerability and Activity Study of Ibudilast in Subjects with Progressive Multiple Sclerosis.”

## Appendix A. R-Program

### R-Program for Table 1

### Row 1 ###
fisher.test(table(nihexample$CTh96YesNo,nihexample$trt.group))
### Row 3–5 ###
*t*.test(nihexample$bpf0~nihexample$trt.group)
*t*.test(nihexample$bpf96~nihexample$trt.group)
### Row 6 ###
library(lme4)
summary(lmer(bpf~trt.group+week+trt.group*week+(1|ID),data=nihexamplelong))

### R-Program for Table 2

hcentr=function(pt,preds,pz,trt, alpha){
s2=var(preds)
if(alpha!=1){
mtrinf=pz/(1-alpha)*((2*pi*s2)^((1-alpha)/2)/sqrt(alpha)*((pt[2])^alpha+(1-pt[2])^alpha)-mean((pnorm(preds[trt==1]))^alpha+(1-pnorm(preds[trt==1]))^alpha))+(1-pz)/(1-alpha)*((2*pi*s2)^((1-alpha)/2)/sqrt(alpha)*((pt[1])^alpha+(1-pt[1])^alpha)-mean((pnorm(preds[trt==0]))^alpha+(1-pnorm(preds[trt==0]))^alpha))}
if(alpha==1){ mtrinf=pz*(mean(pnorm(preds[trt==1])*log(pnorm(preds[trt==1])))+mean(1-pnorm(preds[trt==1])*log(1-pnorm(preds[trt==1])))-pt[2]*log(pt[2])-(1-pt[2])*log(1-pt[2]))+(1-pz)*(mean(pnorm(preds[trt==0])*log(pnorm(preds[trt==0])))+mean(1-pnorm(preds[trt==0])*log(1-pnorm(preds[trt==0])))-pt[1]*log(pt[1])-(1-pt[1])*log(1-pt[1]))}
itma=1-exp(-2*mtrinf)
c(mtrinf,itma)
}
### Row 1 ###
pt=table(nihexample$CTh96YesNo,nihexample$trt.group)[2,]/table(nihexample$trt.group)
myprobit1=glm(CTh96YesNo~trt.group+bpf0+bpf24,family=binomial(link=“probit”), data=nihexample)
row1=round(c(
hcentr(pt,myprobit1$linear.predictors,sum(nihexample$trt.group)/length(nihexample$trt.group),nihexample$trt.group,0.5),
hcentr(pt,myprobit1$linear.predictors,sum(nihexample$trt.group)/length(nihexample$trt.group),nihexample$trt.group,1),
hcentr(pt,myprobit1$linear.predictors,sum(nihexample$trt.group)/length(nihexample$trt.group),nihexample$trt.group,2),
summary(lmer(bpf~trt.group+week+trt.group*week+(1|ID),data=nihexamplelong[nihexamplelong$week==0 |nihexamplelong$week==24, ]))$coefficients[4,5]),4)
### similar codes for other rows ####

### R-Program for Table 3

library(mvtnorm)
library(mvProbit)
####(T, Z)####
table(table(nihexample$CTh96YesNo,nihexample$trt.group)
#####choose varibles: “bpf0”, “bpf24” “bpf48” “bpf72” “bpf96” (1–5) fullmodel1 = mvProbit(cbind(CTh96YesNo,trt.group)~bpf0+bpf24+bpf48+bpf72+bpf96,data=nihexample)
#####model
summary(fullmodel1)
sigma=symMatrix(c(1,fullmodel1$estimate[length(fullmodel1$estimate)],1))
################################################
#mu1=fullmodel1$estimate[1]+fullmodel1$estimate[2]*nihexample$bpf0+fullmodel1$estimate[3]*nihexample$bpf24
#mu2=fullmodel1$estimate[4]+fullmodel1$estimate[5]*nihexample$bpf0+fullmodel1$estimate[6]*nihexample$bpf24
#mu1=fullmodel1$estimate[1]+fullmodel1$estimate[2]*nihexample$bpf0+fullmodel1$estimate[3]*nihexample$bpf24+fullmodel1$estimate[4]*nihexample$bpf48
#mu2=fullmodel1$estimate[5]+fullmodel1$estimate[6]*nihexample$bpf0+fullmodel1$estimate[7]*nihexample$bpf24+fullmodel1$estimate[8]*nihexample$bpf48
#mu1=fullmodel1$estimate[1]+fullmodel1$estimate[2]*nihexample$bpf0+fullmodel1$estimate[3]*nihexample$bpf24+fullmodel1$estimate[4]*nihexample$bpf48+fullmodel1$estimate[5]*nihexample$bpf72
#mu2=fullmodel1$estimate[6]+fullmodel1$estimate[7]*nihexample$bpf0+fullmodel1$estimate[8]*nihexample$bpf24+fullmodel1$estimate[9]*nihexample$bpf48+fullmodel1$estimate[10]*nihexample$bpf72
mu1=fullmodel1$estimate[1]+fullmodel1$estimate[2]*nihexample$bpf0+fullmodel1$estimate[3]*nihexample$bpf24+fullmodel1$estimate[4]*nihexample$bpf48+fullmodel1$estimate[5]*nihexample$bpf72+fullmodel1$estimate[6]*nihexample$bpf96
mu2=fullmodel1$estimate[7]+fullmodel1$estimate[8]*nihexample$bpf0+fullmodel1$estimate[9]*nihexample$bpf24+fullmodel1$estimate[10]*nihexample$bpf48+fullmodel1$estimate[11]*nihexample$bpf72+fullmodel1$estimate[12]*nihexample$bpf96
fullmodel1$estimate
sigma
##################### T|S Z|S############
bs=10000
set.seed(873465)
BT=sample(c(1:length(CTh96YesNo)), size = bs, replace = TRUE) ### bootstrap ID###
BT_U1=matrix(0,bs,2) #### 1 D normal probability
BT_U2=matrix(0,bs,4) ####2 D normal probability
for (i in 1:bs)
{ c=BT[i]
BT_U1[i,1]=1-pnorm(0, mu1[c], 1) ####P(T=1|S)
BT_U1[i,2]=1-pnorm( 0,mu2[c], 1)####P(Z=1|S)
BT_U2[i,1]=pmvnorm(lower=c(0,0),up-per=Inf,mean=c(mu1[c],mu2[c]),sigma)####P(T=1,Z=1|S]
BT_U2[i,2]=pmvnorm(lower=c(0,-Inf),up-per=c(Inf,0),mean=c(mu1[c],mu2[c]),sigma)####P(T=1,Z=0|S]
BT_U2[i,3]=pmvnorm(lower=c(-Inf,0),upper=c(0,Inf),mean=c(mu1[c],mu2[c]),sigma)####P(T=0,Z=1|S]
BT_U2[i,4]=pmvnorm(lower=-Inf,upper=c(0,0),mean=c(mu1[c],mu2[c]),sigma)####P(T=0,Z=0|S]}
################################
alfa=0.5
p1=mean((BT_U1[,1]*BT_U1[,2])^alfa+((1-BT_U1[,1])*BT_U1[,2])^alfa+(BT_U1[,1]*(1-BT_U1[,2]))^alfa+((1-BT_U1[,1])*(1-BT_U1[,2]))^alfa)
p2=mean(BT_U2[,1]^alfa+BT_U2[,2]^alfa+BT_U2[,3]^alfa+BT_U2[,4]^alfa)
I_alfa=1/(1-alfa)*(p1-p2)
IM_alfa=1-exp(-2*I_alfa)
I_alfa
IM_alfa
###################################
alfa=2
p1=mean((BT_U1[,1]*BT_U1[,2])^alfa+((1-BT_U1[,1])*BT_U1[,2])^alfa+(BT_U1[,1]*(1-BT_U1[,2]))^alfa+((1-BT_U1[,1])*(1-BT_U1[,2]))^alfa) 
p2=mean(BT_U2[,1]^alfa+BT_U2[,2]^alfa+BT_U2[,3]^alfa+BT_U2[,4]^alfa)
I_alfa=1/(1-alfa)*(p1-p2)
IM_alfa=1-exp(-2*I_alfa)
I_alfa
IM_alfa
######## alfa=1#####################
p1=-mean(BT_U1[,1]*log(BT_U1[,1])+(1-BT_U1[,1])*log(1-BT_U1[,1]))
p2=-mean(BT_U1[,2]*log(BT_U1[,2])+(1-BT_U1[,2])*log(1-BT_U1[,2]))
p3=mean(BT_U2[,1]*log(BT_U2[,1])+BT_U2[,2]*log(BT_U2[,2])+BT_U2[,3]*log(BT_U2[,3]) +BT_U2[,4]*log(BT_U2[,4]))
I=p1+p2+p3
IM=1-exp(-2*I)
